# BACE2 Promotes Bladder Cancer Chemoresistance Through Proteolytic Activation of JAG1‐Notch Signaling

**DOI:** 10.1002/cai2.70073

**Published:** 2026-08-18

**Authors:** Zekun Li, Sanxiang Li, Zhenyu Liu, Yulin Sun, Yun Zhang

**Affiliations:** ^1^ State Key Laboratory of Molecular Oncology National Cancer Center/National Clinical Research Center for Cancer/Cancer Hospital, Chinese Academy of Medical Sciences and Peking Union Medical College Beijing China; ^2^ Beijing Key Laboratory of Urologic Cancer Cell and Gene Therapy National Cancer Center/National Clinical Research Center for Cancer/Cancer Hospital, Chinese Academy of Medical Sciences and Peking Union Medical College Beijing China; ^3^ Department of Urology Inner Mongolia Autonomous Region People's Hospital Hohhot Inner Mongolia Autonomous Region China

**Keywords:** B‐secretase 2, drug resistance, Notch pathway, urinary bladder neoplasms

## Abstract

**Background:**

Chemoresistance to platinum‐based regimens, primarily gemcitabine plus cisplatin, remains a major obstacle in the treatment of advanced bladder cancer. Identifying the underlying molecular mechanisms is critical for developing effective therapeutic strategies to improve clinical outcomes.

**Methods:**

Integrative analysis of transcriptomic profiles from chemoresistant bladder cancer models and clinical data sets from The Cancer Genome Atlas was performed to identify candidate resistance drivers. The biological function of the candidate gene was evaluated in vitro using bladder cancer cell lines. Mechanistic investigations, including transcriptomic analyses and immunoprecipitation, were conducted to elucidate downstream signaling. The therapeutic potential of targeting this axis was assessed in vitro and in an in vivo xenograft model using a small‐molecule inhibitor combined with chemotherapy.

**Results:**

We identified β‐secretase 2 (BACE2) as a candidate mediator of cisplatin resistance. *BACE2* is significantly upregulated in gemcitabine‐ and cisplatin‐resistant models, including patient‐derived organoids. Its elevated baseline expression correlates with advanced pathologic stage, poor prognosis, and diminished clinical benefit from platinum‐based therapy. Functionally, BACE2 promotes resistance to both gemcitabine and cisplatin. Mechanistically, BACE2 interacts with Jagged1 (JAG1) and promotes the generation of a soluble JAG1 fragment in a catalytic activity‐dependent manner. Mutation of the catalytic Asp303 residue markedly reduced JAG1 fragment generation and restored gemcitabine sensitivity, supporting a functional requirement for BACE2 enzymatic activity in this process. This fragment is associated with Notch signaling activation through autocrine and paracrine mechanisms, which concomitantly induces endogenous JAG1 expression, establishing a positive feedback loop that sustains chemoresistance. Pharmacologic inhibition of β‐secretase activity with verubecestat disrupted JAG1 cleavage, reduced downstream Notch signaling and enhanced chemotherapy efficacy in vitro without inducing apparent cytotoxicity alone. Furthermore, combination treatment with verubecestat and gemcitabine produced stronger tumor growth inhibition than either monotherapy in vivo.

**Conclusions:**

These results define a novel BACE2‐JAG1‐Notch signaling axis that contributes to bladder cancer chemoresistance. Importantly, our findings identify BACE2 as a potential prognostic biomarker and a therapeutically actionable target to improve clinical outcomes in this disease.

## Graphical abstract

Proposed model of β‐secretase 2 (BACE2)‐mediated chemoresistance in bladder cancer. In BACE2‐low bladder cancer cells (left), the Notch signaling axis remains silenced, rendering cells highly sensitive to chemotherapy. In BACE2‐high cells (right), BACE2 mediates cleavage of transmembrane Jagged1 (JAG1) to release soluble JAG1 (sJAG1). Free sJAG1 acts through autocrine and paracrine pathways to bind Notch receptors, triggering receptor cleavage and the release of the Notch intracellular domain (NICD). Upon nuclear translocation, NICD activates downstream target genes, including HES1 and JAG1, forming a positive feedback loop that amplifies Notch signaling, ultimately promoting tumor cell proliferation, survival, and resistance to gemcitabine (Gem) and cisplatin (Cis). (The graphical abstract image was Created in BioRender. Lee, G. (2026) https://BioRender.com/pdss9sw)

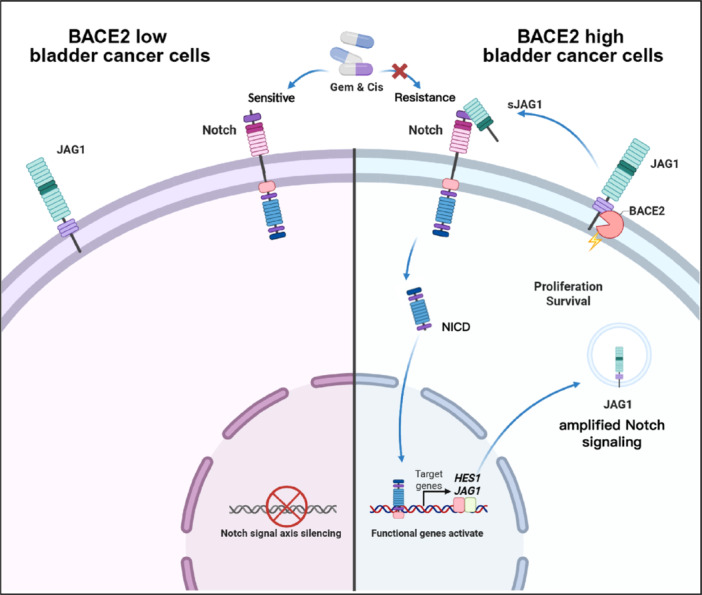

**Abbreviations**
BACE2: β‐secretase 2
BLCA: bladder urothelial carcinoma
DAPT: *N*‐[*N*‐(3,5‐difluorophenacetyl)‐l‐alanyl]‐*S*‐phenylglycine *t*‐butyl ester
DMSO: dimethyl sulfoxide
EV: empty vector
GAPDH: glyceraldehyde‐3‐phosphate dehydrogenase
GEO: Gene Expression Omnibus
HES1: Hes Family bHLH Transcription Factor 1
IHC: immunohistochemistry
IP: Immunoprecipitation
JAG1: Jagged1
PDOs: patient‐derived organoids
qPCR: quantitative real‐time polymerase chain reaction
RNA‐seq: RNA‐sequencing
TCGA: the cancer genome atlas

## 1 Introduction

Bladder cancer is one of the most common malignancies of the urinary tract and is marked by a high rate of recurrence and progression [1]. Chemotherapy remains an important component of treatment across multiple disease settings [2]. In muscle‐invasive bladder cancer, cisplatin‐based neoadjuvant chemotherapy improves survival and is part of standard management. In advanced urothelial carcinoma, platinum‐based regimens, particularly gemcitabine plus cisplatin, continue to play a central role despite recent advances in immunotherapy and antibody–drug conjugates [3, 4]. However, a substantial proportion of patients show limited initial response or eventually develop disease progression [5], highlighting chemoresistance as a major obstacle to durable benefit.

The mechanisms underlying chemoresistance in bladder cancer are complex and remain incompletely understood. Previous studies have implicated multiple contributing processes, including genomic instability, molecular subtype‐specific programs, altered DNA damage repair, metabolic adaptation, and cell‐state plasticity [6, 7, 8, 9, 10]. Despite these advances, few resistance mechanisms have translated into clinically useful biomarkers or therapeutic targets. This remains a significant unmet need, particularly because platinum‐ and gemcitabine‐based chemotherapy is still widely used in routine clinical care.

β‐secretase 2 (BACE2) is a β‐secretase family aspartic protease best known for its role in neural biology and amyloid precursor protein processing [11]. More recently, evidence has emerged suggesting that BACE2 may have functions beyond the nervous system. Elevated BACE2 expression has been reported in several tumor types and has been associated with adverse clinical outcomes. In addition, recent work has linked BACE2 to proteolytic regulation of cancer‐associated metabolic pathways, raising the possibility that it may contribute to tumor progression in nonneural tissues [12, 13, 14, 15, 16, 17]. However, whether BACE2 has a role in cancer therapy response has not been explored.

## 2 Methods

### 2.1 Bioinformatics and Clinical Database

Publicly available RNA‐sequencing (RNA‐seq) data sets of gemcitabine‐resistant (GemR) bladder cancer cell lines (GSE190636 and GSE210954), cisplatin‐resistant patient‐derived organoids (PDOs; GSE192575), and cisplatin‐resistant cell lines (GSE33482, GSE108214, and GSE148003) were obtained from the Gene Expression Omnibus (GEO). When analyzing GSE192575 scRNA‐seq data set, malignant epithelial cells were extracted according to tumor marker expression, and *BACE2* expression was compared between cisplatin‐resistant and cisplatin‐sensitive groups. Fold change (FC) was calculated based on the mean normalized *BACE2* expression between resistant and sensitive tumor cells.

Clinical and transcriptomic data for bladder urothelial cancer (BLCA), lung adenocarcinoma, and cervical squamous cell carcinoma were downloaded from The Cancer Genome Atlas (TCGA). Baseline *BACE2* expression across cancer cell lines was retrieved from the Cancer Cell Line Encyclopedia. Patient survival prognostics and treatment responsiveness data were evaluated using the GEPIA3 web server. Unless otherwise specified, patients were stratified into *BACE2*‐high and *BACE2*‐low groups using the median *BACE2* expression level as the cutoff. For pathway activity comparisons in TCGA‐BLCA, the top 25% and bottom 25% of patients ranked by *BACE2* expression were defined as *BACE2*‐high and *BACE2*‐low groups, respectively. Differentially expressed gene analysis was defined using cutoff criteria of *p* < 0.05 and |log_2_ FC| > 1. Gene Set Enrichment Analysis (GSEA) and Gene Ontology (GO) enrichment were performed, with pathway activation defined by a Normalized Enrichment Score > 1 and *p* < 0.05. Spearman's rank correlation was utilized to analyze gene coexpression in the TCGA BLCA data set.

### 2.2 Cell Culture and Reagents

Human bladder cancer cell lines (T24, 5637, and HT1376) were obtained from the Institute of Basic Medical Sciences, Chinese Academy of Medical Sciences (Beijing, China), and cultured according to standard protocols. Gemcitabine, cisplatin, the small‐molecule β‐secretase inhibitor verubecestat, and the Notch pathway inhibitor *N*‐[*N*‐(3,5‐difluorophenacetyl)‐L‐alanyl]‐*S*‐phenylglycine *t*‐butyl ester (DAPT) were purchased from MedChemExpress (Monmouth Junction, NJ, USA). All drugs were dissolved in dimethyl sulfoxide (DMSO; Sigma‐Aldrich, St. Louis, MO, USA) or appropriate solvents for in vitro assays.

### 2.3 Plasmid Construction and Lentiviral Transduction

To establish stable cell lines, T24 cells were transfected with an empty vector (EV) or a *BACE2* overexpression construct. Conversely, 5637 cells were transduced with lentiviruses expressing a nontargeting short hairpin RNA (shRNA) (shControl) or *BACE2*‐specific shRNAs (*BACE2* KD). The specific shRNA target sequences were as follows: shControl (5′‐CAACAAGATGAAGAGCACC‐3′), sh‐*BACE2‐1* (5′‐GCCTTTCTCAACAGAGGTGT‐3′), and sh‐*BACE2‐2* (5′‐CCTTGGTCTTACTTCCCTAAA‐3′). For rescue and epistatic experiments, Jagged1 (*JAG1*) was knocked down in T24 *BACE2*‐OE cells using a *JAG1*‐specific shRNA (target sequence: 5′‐GTCAGAATTGTGACATAAA‐3′), while *JAG1* was overexpressed in HT1376 *BACE2*‐KD cells. Transfection and knockdown (KD) efficiencies were rigorously validated via Western blotting and quantitative real‐time polymerase chain reaction (qPCR).

### 2.4 Cell Viability and Colony Formation Assays

Cellular sensitivity to chemotherapeutic agents (gemcitabine and cisplatin) and verubecestat was assessed using the Cell Counting Kit‐8 (CCK‐8) assay. Cells were seeded into 96‐well plates at a density of 5000 cells/well and treated with the indicated compounds for 48 h before absorbance measurement. For colony formation assays, cells were seeded in 6‐well plates at a density of 500 cells/well and cultured with varying concentrations of verubecestat (0.1, 1, and 10 μM) or DMSO control for 10–14 days. Colonies were subsequently fixed, stained with crystal violet, and quantified.

### 2.5 Conditioned Medium Assay

To evaluate paracrine signaling, cell culture supernatants were collected from T24 EV and T24 *BACE2*‐OE cells. Naive T24 recipient cells were pre‐seeded in 96‐well plates and cultured with conditioned medium derived from T24‐EV or T24‐*BACE2*‐OE cells. Where indicated, recipient cells were treated with the Notch pathway inhibitor DAPT at 10 μM during conditioned medium exposure. After conditioned medium treatment, cells were exposed to gemcitabine for 48 h, followed by the CCK‐8 analysis. Recipient cells were also harvested for qPCR and Western blot analysis of JAG1 and Hes Family bHLH Transcription Factor 1 (HES1) expression.

### 2.6 Quantitative Real‐Time PCR

Total RNA was extracted from cultured cells, reverse‐transcribed into complementary DNA, and subjected to qPCR analysis. Relative messenger RNA expression levels of *BACE2*, *JAG1*, and *HES1* were quantified, with glyceraldehyde‐3‐phosphate dehydrogenase (*GAPDH*) serving as the internal reference gene. The specific primer sequences used were as follows:

*JAG1*: 5′‐CCTGTGAAGTGATTGACAGC‐3′ (forward) and 5′‐CGTTGGAGGAAATATACCGC3′ (reverse);

*HES1*: 5′‐TGCCAGCTGATATAATGGAG‐3′ (forward) and 5′‐CTTTGATGACTTTCTGTGCTC‐3′ (reverse);

*BACE2*: 5′‐TGCCTGGGATTAAATGGAATGG‐3′ (forward) and 5′‐CAGGGAGTCGAAGAAGGTCTC‐3′ (reverse);

*GAPDH*: 5′‐GTCTCCTCTGACTTCAACAGCG‐3′ (forward) and 5′‐ACCACCCTGTTGCTGTAGCCAA‐3′ (reverse).

All qPCR assays were performed with three technical replicates per sample.

### 2.7 Western Blotting and Immunoprecipitation (IP)

Total protein was extracted and normalized before separation via SDS‐PAGE. Membranes were probed with primary antibodies against BACE2 (Abcam, ab27048), JAG1 (Cell Signaling Technology, #70109), HES1 (Cell Signaling Technology, #11988), Notch intracellular domain (NICD) (Cell Signaling Technology, #4147), and GAPDH (Proteintech, 60004‐1‐Ig). For IP assays, endogenous BACE2 was immunoprecipitated from T24 *BACE2*‐OE and parental 5637 cell lysates using a BACE2‐specific antibody. Normal immunoglobulin G served as the negative control, and total cell lysates (input) served as the positive control. Immunocomplexes were subsequently blotted for JAG1 to confirm physical interaction. The working dilutions used were according to the manufacturers' recommended optimal conditions.

### 2.8 Protein–Protein Docking Prediction

The three‐dimensional binding interaction between BACE2 and JAG1 was modeled using global range molecular matching rigid docking. Binding affinity and interaction interfaces were evaluated based on docking conformations, with optimal binding defined by the lowest binding energy (−18.9 kcal/mol) and the presence of critical intermolecular hydrogen bonds (lengths ranging from 1.5 to 3.5 Å).

### 2.9 In Vivo Xenograft Model

All experiments involving animals were conducted in accordance with institutional guidelines and applicable regulations for animal welfare and laboratory animal care. Seven‐week‐old male Bagg albino c (BALB/c) nude mice were subcutaneously implanted with 5637 cells (4 × 10^6^ cells/mouse suspended in 100 μL of a 1:1 PBS/Matrigel mixture) into the right axillary region. Tumor volumes were measured using digital calipers and calculated as Volume = (length × width^2^)/2. When tumors reached an average volume of approximately 50 mm^3^, mice were randomly assigned to four treatment groups (*n* = 8 mice per group): vehicle control, gemcitabine alone (70 mg/kg), verubecestat alone (3 mg/kg), and gemcitabine plus verubecestat. Treatments were administered by intraperitoneal injection once every 3 days for a total of four cycles. Tumor volume and mouse body weight were monitored throughout the treatment period. At the experimental endpoint, mice were euthanized, and tumors were excised, weighed, photographed, and processed for histological and immunohistochemical (IHC) analyses.

### 2.10 Histology and Immunohistochemistry (IHC)

Excised xenograft tumors were formalin‐fixed and paraffin‐embedded. Tissue sections were subjected to hematoxylin and eosin (H&E) staining to evaluate general tumor morphology. IHC staining was performed to detect the expression of Ki‐67, JAG1, and HES1. Positive staining was visualized using 3,3′‐diaminobenzidine, and expression levels were quantitatively evaluated using the continuous H‐score methodology.

### 2.11 Statistical Analysis

Data are presented as the mean ± standard deviation (SD) unless otherwise indicated. For in vitro experiments, at least three independent biological replicates were performed unless stated otherwise. Technical replicates were averaged before statistical comparison and were not treated as independent biological replicates. Data normality was assessed using the Shapiro–Wilk test, and homogeneity of variance was evaluated using Levene's test. For comparisons between two groups, unpaired two‐tailed Student's *t* tests were used when data met the assumptions of normality and equal variance; otherwise, the Mann–Whitney *U* test was applied. For comparisons among multiple groups, one‐way analysis of variance (ANOVA) followed by Tukey's post hoc test was used for normally distributed data with equal variance; otherwise, the Kruskal–Wallis test followed by Dunn's multiple‐comparison test was applied. Tumor growth curves were analyzed using two‐way repeated‐measures ANOVA, with treatment group, time, and group‐by‐time interaction included in the model, followed by appropriate post hoc multiple‐comparison tests. For transcriptomic analyses, multiple‐testing correction was performed using the Benjamini–Hochberg false discovery rate method where applicable. A *p* value < 0.05 was considered statistically significant.

## 3 Results

### 3.1 Identification of *BACE2* as a Candidate Driver of Chemoresistance in Bladder Cancer

**Figure 1:**
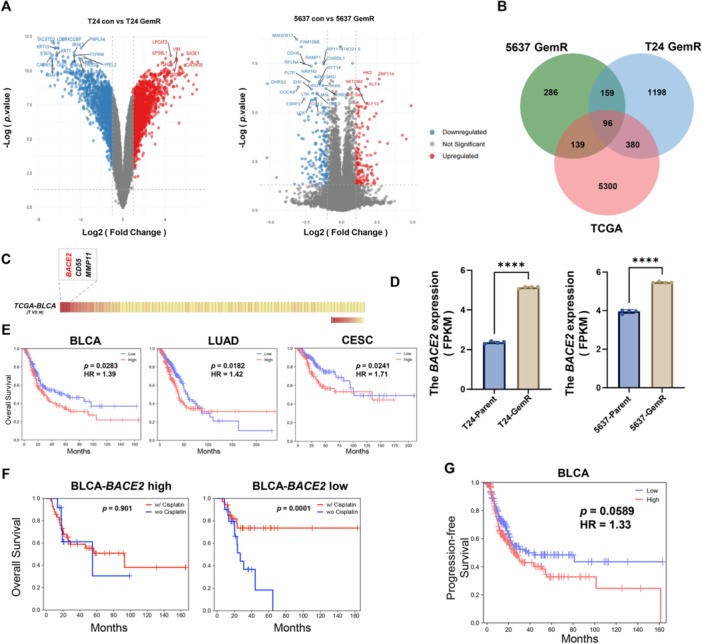
Identification of BACE2 as a candidate chemoresistance–related gene in bladder cancer. (A) Volcano plot depicting differentially expressed genes (DEGs) between Gemcitabine‐resistant (GemR) cells and parental cells (T24 and 5637 cell lines; GSE190636 and GSE210954 data sets). Blue dots represent downregulated genes, and red dots represent upregulated genes, using significance cutoffs of *p* < 0.05 and |log_2_ FC| > 1. (B) Venn diagram illustrating the number of genes: (1) upregulated in GemR T24 and 5637 cell lines and (2) linked to poor disease progression in the TCGA BLCA data set (cutoff: HR > 1.2, *p* < 0.05). (C) Heatmap showing differential expression of the 96 overlapping genes (from Figure B) between tumor tissues and adjacent nontumor tissues in the TCGA BLCA data set. The intensity of red corresponds to the magnitude of gene overexpression in tumor tissues. (D) Comparison of BACE2 expression levels in sequencing data. The left panel shows BACE2 sequencing results in T24 cells, and the right panel shows those in 5637 cells (*n* = 4). (E) Kaplan–Meier survival curves showing the association between BACE2 expression and overall survival in TCGA‐BLCA. Lung adenocarcinoma and cervical squamous cell carcinoma were included as exploratory cohorts based on preliminary GEPIA3 survival analyses. Data were retrieved from GEPIA3. Patients were divided into BACE2‐high and BACE2‐low groups using the median BACE2 expression level as the cutoff. (F) Comparison of cisplatin responsiveness between patients with high and low BACE2 expression in the TCGA BLCA data set. Data were retrieved from GEPIA3. Patients were divided into BACE2‐high and BACE2‐low groups using the median BACE2 expression level as the cutoff. (G) Progression‐free survival curves demonstrating the effect of BACE2 expression on patient prognosis across the TCGA BLCA data set. Data were retrieved from GEPIA3. Patients were divided into BACE2‐high and BACE2‐low groups using the median BACE2 expression level as the cutoff. Data are presented as mean ± SD. Statistical significance was determined by unpaired two‐tailed *t* tests (D). ****p ≤ 0.0001. BACE2, β‐secretase 2; BLCA, bladder urothelial carcinoma; CESC, cervical squamous cell carcinoma and endocervical adenocarcinoma; FPKM, fragments per kilobase of transcript per million mapped reads; HR, hazard ratio; LUAD, lung adenocarcinoma; TCGA, the cancer genome atlas.

To systematically identify molecular drivers of gemcitabine resistance in bladder cancer, we interrogated publicly available RNA‐seq data sets from the GEO database (GSE190636 and GSE210954) for gemcitabine‐resistant bladder cancer cell lines. These data sets comprise transcriptomic profiles of two well‐established bladder cancer cell lines, T24 and 5637, each with matched GemR derivatives and their corresponding drug‐sensitive parental controls. Differential expression analysis revealed extensive transcriptional reprogramming associated with acquired resistance. In GemR T24 cells, 1833 genes were significantly upregulated and 1584 were downregulated relative to parental cells. Similarly, resistant 5637 cells exhibited 1005 upregulated and 1008 downregulated genes in resistant 5637 cells, compared with their respective drug‐sensitive parental lines (Figure 1A). To prioritize clinically relevant candidates and with therapeutic potential, we intersected genes upregulated in both resistant cell lines with bladder cancer prognosis–associated genes (hazard ratio > 1.2) derived from the TCGA database. This integrative filtering strategy yielded 96 overlapping genes (Figure 1B), enriching for resistance‐associated transcripts linked to adverse clinical outcome.

We next examined the expression of these 96 candidates in TCGA bladder cancer tissues compared with adjacent normal tissues. Among them, *BACE2* emerged as the most significantly upregulated gene in BLCA (Figure 1C). In GemR models, *BACE2* expression was increased by approximately 2.2‐fold in T24‐GemR cells compared with T24‐Parent cells (log_2_ FC = 1.21, *p* < 0.0001), and by approximately 1.7‐fold in 5637‐GemR cells compared with 5637‐Parent cells (log_2_ FC = 0.76, *p* < 0.0001) (Figure 1D). We further assessed the prognostic relevance of *BACE2* in TCGA cohorts. In BLCA, high *BACE2* expression was associated with poorer overall survival, supporting its clinical relevance in bladder cancer (Figure 1E). An exploratory pan‐cancer survival analysis revealed similar adverse prognostic associations with *BACE2* expression in lung adenocarcinoma and cervical squamous cell carcinoma (Figure 1E). These data suggest that the prognostic relevance of *BACE2* may extend beyond BLCA in selected epithelial malignancies, although the mechanistic analyses in this study were focused specifically on bladder cancer chemoresistance.

In the BLCA cohort, stratification by *BACE2* expression further showed that the clinical benefit associated with chemotherapy was more evident in the *BACE2*‐low group, whereas this association was attenuated in patients with high *BACE2* expression (Figure 1F). Consistently, retrospective analysis of recurrence data from the TCGA‐BLCA cohort showed that high *BACE2* expression was significantly associated with an increased risk of bladder cancer recurrence (Figure 1G). Given the predominance of gemcitabine plus cisplatin regimens in advanced bladder cancer, these clinical associations are consistent with a potential role for *BACE2* in chemoresistance.

### 3.2 BACE2 Functionally Promotes Gemcitabine and Cisplatin Resistance in Bladder Cancer

**Figure 2:**
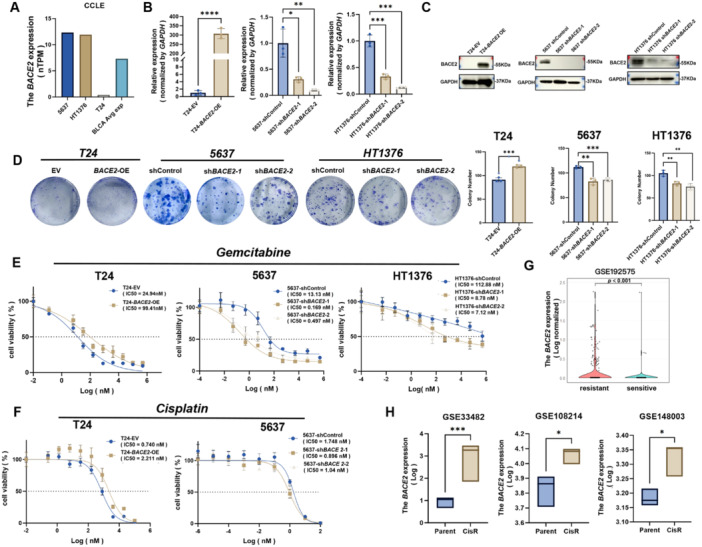
BACE2 is highly expressed in chemoresistant bladder cancer cells and promotes chemoresistance. (A) Bar plot showing BACE2 expression levels (nTPM) across diverse cancer cell lines. Data were obtained from the Cancer Cell Line Encyclopedia database. (B) Quantitative real‐time polymerase chain reaction (qPCR) analysis to determine BACE2 relative expression. The left panel presents results for T24 cells, the middle panel for 5637 cells, and the right panel for HT1376 cells. GAPDH served as the internal reference gene. (C) Western blot analysis to detect BACE2 protein expression. The left panel shows results for T24 cells, the middle panel for 5637 cells, and the right panel for HT1376 cells. GAPDH was used as the loading control. (D) Colony formation assay to assess cell clonogenic potential. The left panel displays representative photographs of colony‐forming plates, and the right panel shows the quantification of colony numbers (*n* = 3). (E) The Cell Counting Kit‐8 (CCK‐8) assay to evaluate the sensitivity of cells to Gemcitabine. Cells were seeded in 96‐well plates at a density of 5000 cells per well, after which they were treated with Gemcitabine for 48 h. (F) CCK‐8 assay to assess the sensitivity of cells to cisplatin. Cells were seeded in 96‐well plates at a density of 5000 cells per well, followed by cisplatin treatment for 48 h. (G) Analysis of BACE2 expression levels in tumor cells. Red represents the cisplatin‐resistant group, and blue represents the cisplatin‐sensitive group. Data were obtained from the GEO database (GSE192575) (resistant: *n* = 1392 tumor cells from one resistant sample; sensitive: *n* = 1590 tumor cells from one sensitive sample; mean normalized expression: resistant versus sensitive; log_2_ FC = 3.73; *p* < .0001). (H) Analysis of BACE2 expression levels (Log(count)) in cisplatin‐resistant cell lines (yellow) and parental (cisplatin‐sensitive) cell lines (blue). Data were obtained from the GEO database (GSE33482, GSE108214, and GSE148003). Data are presented as mean ± SD. Statistical significance was determined by unpaired two‐tailed *t* tests (B, D, H). *p ≤ 0.05; **p ≤ 0.01; ***p ≤ 0.001; ****p ≤ 0.0001. BACE2, β‐secretase 2; BLCA, bladder urothelial carcinoma; CCLE, cancer cell line encyclopedia; EV, empty vector; GAPDH, glyceraldehyde‐3‐phosphate dehydrogenase; GEO, gene expression omnibus; nTPM, normalized transcripts per million; OE, overexpressing; SD, standard deviation.

To determine whether BACE2 actively drives chemoresistance, we evaluated the intrinsic drug sensitivity of parental bladder cancer cells following *BACE2* modulation. shRNA‐mediated KD of *BACE2* in high‐expressing human bladder cancer cell lines (HT1376 and 5637) resulted in a modest but significant reduction in proliferative capacity. Although reduced proliferation is often expected to lessen the sensitivity to cytotoxic agents, *BACE2*‐KD cells exhibited markedly increased sensitivity to gemcitabine (Figure 2A–E). These results suggest that chemo‐sensitizing effect of *BACE2* KD cannot be explained simply by altered proliferation, but instead reflects a functional role for BACE2 in protecting bladder cancer cells from chemotherapy‐induced cytotoxic stress. Conversely, enforced expression of *BACE2* in the low‐expressing bladder cancer cell line T24 enhanced cellular proliferation and significantly reduced gemcitabine sensitivity (Figure 2A–E). Together, these gain‐ and loss‐of‐function experiments establish a functional role for BACE2 in promoting chemoresistance in bladder cancer cells.

We next assessed whether BACE2 similarly influences cisplatin responsiveness. Consistent with our findings in gemcitabine‐treated cells, *BACE2* overexpression conferred increased resistance to cisplatin, whereas *BACE2* KD significantly sensitized bladder cancer cells to cisplatin‐induced cytotoxicity (Figure 2F). This is consistent with elevated *BACE2* expression in cisplatin‐resistant derivatives compared with their parental cell lines across multiple cancer cell line models. To extend these observations to more clinically relevant models, we analyzed an independent GEO data set (GSE192575) comprising cisplatin‐sensitive and cisplatin‐resistant bladder cancer PDOs (Figure 2G). *BACE2* expression was significantly elevated in cisplatin‐resistant cancer cells compared with their sensitive counterparts in the other cisplatin‐resistant data sets (Figure 2H).

### 3.3 BACE2 Promotes Chemoresistance Through Activation of JAG1‐Notch Signaling

**Figure 3:**
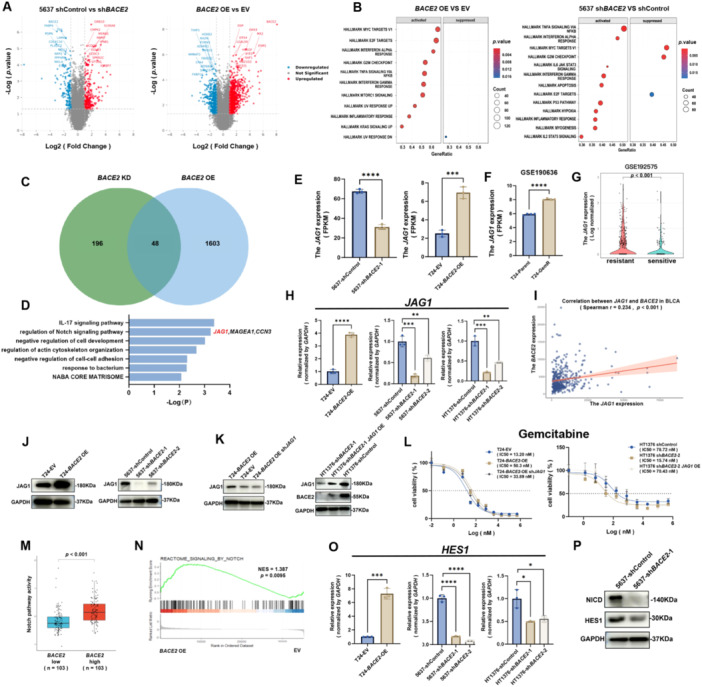
BACE2 upregulates JAG1 expression and activates Notch signaling to promote chemoresistance in bladder cancer. (A) Volcano plots of differentially expressed genes (DEGs) comparing 5637 BACE2‐knockdown (KD) cells versus 5637 nontargeting shRNA (shControl) controls (left panel), and T24 BACE2 overexpression cells versus T24 EV controls (right panel). Blue and red dots denote significantly downregulated and upregulated DEGs (*p* < 0.05 and |log_2_ FC| > 1), respectively. (B) Gene Set Enrichment Analysis (GSEA) showing significantly enriched Hallmark pathways in T24 BACE2 OE versus T24 EV cells (left panel) and 5637 BACE2 KD versus 5637 shControl cells (right panel). Pathways were considered statistically significant at *p* < 0.05. (C) Venn diagram showing the overlap between significantly downregulated genes in 5637 BACE2 KD cells (green circle) and significantly upregulated genes in T24 BACE2 OE cells (blue circle). A total of 48 common genes were identified in the intersection. (D) Gene Ontology (GO) enrichment analysis of the 48 overlapping genes (from Figure C). GO terms were considered significantly enriched at *p* < 0.05. (E) JAG1 expression levels (expressed as fragments per kilobase of transcript per million mapped reads, FPKM) from RNA sequencing data. Left panel: 5637 BACE2 KD versus 5637 shControl cells; right panel: T24 BACE2 OE versus T24 EV cells (*n* = 3). (F) JAG1 expression levels (*n* = 4) from the Gene Expression Omnibus (GEO) database (accession no. GSE190636). (G) JAG1 expression in tumor cells. Red indicates the drug‐resistant group, and blue indicates the drug‐sensitive group. Data were obtained from the GEO database (accession no. GSE192575). (H) Quantitative real‐time polymerase chain reaction (qPCR) analysis of JAG1 expression in T24 (left panel), 5637 (middle panel), and HT1376 (right panel) cells (*n* = 3 technical replicates). Glyceraldehyde‐3‐phosphate dehydrogenase (GAPDH) was used as the internal reference gene. Unpaired *t* tests were performed to compare each experimental group with its respective control. (I) Spearman's rank correlation analysis of JAG1 and BACE2 expression in the TCGA BLCA data set. (J) Western blot analysis of JAG1 protein expression in T24 (left panel) and 5637 (right panel) cells after BACE2 modulation. GAPDH was used as the loading control. (K) Western blot analysis validating JAG1 KD efficiency in JAG1‐silenced T24 BACE2‐OE cells and JAG1 overexpression efficiency in BACE2 KD cells in the HT1376 cell line. GAPDH was used as the loading control. (L) CCK‐8 assay to determine the sensitivity to Gemcitabine. Cells were seeded in 96‐well plates at 5000 cells/well, and Gemcitabine treatment was initiated thereafter for 48 h. (M) Notch signaling pathway activity in the TCGA BLCA data set, comparing BACE2‐high‐expression (top 25%) and low‐expression (bottom 25%) groups. (N) GSEA to assess the activation status of Notch‐related signaling pathways. Pathways were considered significantly activated if they met the criteria of Normalized Enrichment Score > 1 and *p* < 0.05. (O) qPCR analysis of HES1 expression in 5637, HT‐1376, and T24 cells (*n* = 3 technical replicates). GAPDH served as the internal reference gene. (P) Western blot analysis of HES1 and NICD protein expression in 5637‐shBACE2 versus 5637‐shControl cells. GAPDH served as the loading control. Data are presented as mean ± SD. Statistical significance was determined by unpaired two‐tailed *t* tests (E, F, H, O). *p ≤ 0.05; **p ≤ 0.01; ***p ≤ 0.001; ****p ≤ 0.0001. BACE2, β‐secretase 2; BLCA, Bladder Urothelial Carcinoma; CCK‐8, cell counting kit‐8; EV, empty vector; FC, fold change; FPKM, fragments per kilobase of transcript per million mapped reads; HES1, Hes Family bHLH Transcription Factor 1; JAG1, Jagged1; NICD, Notch intracellular domain; OE, overexpressing; SD, standard deviation; TCGA, The Cancer Genome Atlas.

To unravel the molecular mechanisms driving BACE2‐mediated chemoresistance, we performed bulk RNA‐seq following genetic modulation of *BACE2*. Transcriptomic profiling was conducted in *BACE2*‐overexpressing (OE) T24 cells and *BACE2*‐KD 5637 cells, alongside their respective control counterparts. *BACE2* dysregulation‐induced broad transcriptional reprogramming, with 1721 genes upregulated and 478 downregulated (|log_2_ FC|> 1, *p* < 0.05), whereas *BACE2* KD resulted in 303 upregulated and 168 downregulated genes (Figure 3A). GSEA revealed significant enrichment of MYC signaling and cell‐cycle‐associated pathways upon increased *BACE2* expression, consistent with its observed role in promoting cell proliferation (Figure 3B).

To identify downstream effectors potentially mediating chemoresistance, we intersected genes upregulated by *BACE2* overexpression with those downregulated upon *BACE2* KD, yielding 48 candidates. GO analysis of this gene set revealed significant enrichment of biological processes related to extracellular matrix remodeling, interleukin‐17 (IL‐17) signaling pathway, and Notch pathway regulation (Figure 3C,D). Although extracellular matrix‐remodeling and IL‐17‐associated programs likely reflect broader effects on tumor progression and microenvironmental adaptation, Notch signaling has been directly linked to chemotherapy resistance in bladder cancer [18]. We therefore focused on the Notch pathway as the most plausible mechanistic link between *BACE2* and the resistant phenotype.

Among the Notch pathway–related genes enriched in our analysis, *JAG1* (Jagged1) emerged as a prominent candidate (Figure 3D). Notably, *JAG1* expression is positively correlated with *BACE2* levels: it was upregulated in *BACE2*‐OE cells, downregulated upon *BACE2* KD, and elevated in GemR T24 cells as well as in cisplatin‐resistant PDOs (Figure 3E–J). Importantly, *JAG1* KD in *BACE2*‐OE T24 cells reversed the increased gemcitabine resistance. Furthermore, re‐expression of *JAG1* in *BACE2*‐KD cell lines restored the resistance of cancer cells to gemcitabine. These results support a functional role for JAG1‐Notch signaling in BACE2‐driven chemoresistance (Figure 3K,L).

Analysis of the TCGA bladder cancer cohort further demonstrated a strong positive correlation between *BACE2* expression and *JAG1*‐Notch signaling. Patients with high *BACE2* expression exhibited significantly elevated *JAG1* levels and increased Notch pathway activity (Figure 3M,N). Consistently, *HES1* expression and NICD protein levels mirrored BACE2 expression in genetically modified cell lines (Figure 3O,P).

### 3.4 BACE2 Promotes JAG1 Processing and Notch Activation in a Catalytic Activity‐Dependent Manner

While our data positioned *JAG1*‐Notch signaling downstream of *BACE2*, the precise molecular mechanism linking this protease to pathway activation remained undefined. Previous studies have shown that BACE1, a homolog of BACE2, binds JAG1 and mediates its S2 cleavage to generate a soluble ~25 kDa extracellular fragment (sJAG1) [19, 20]. This raised the possibility that BACE2 may similarly process JAG1 to modulate Notch signaling.

**Figure 4:**
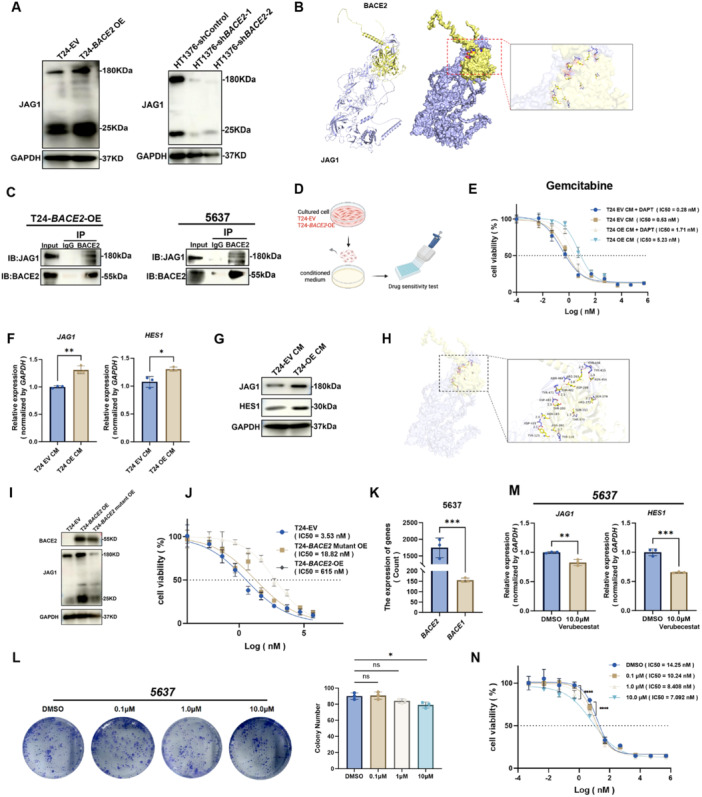
BACE2 physically interacts with and cleaves JAG1, leading to Notch pathway activation and chemoresistance in bladder cancer. (A) Western blot analysis to investigate BACE2‐mediated cleavage of JAG1. The upper band (180 kDa) represents uncleaved full‐length JAG1, while the lower band (25 kDa) corresponds to the cleaved soluble JAG1 (sJAG1). GAPDH was used as the loading control. (B) Three‐dimensional model of BACE2 (yellow) in complex with JAG1 (purple), generated by GRAMM rigid docking. Red dashed lines represent hydrogen bonds (lengths in Å), with shorter bonds (1.5–3.5 Å) indicating more critical residues at the interface. The top‐ranked conformation shows optimal binding (binding energy: −18.9 kcal/mol), supporting a stable interaction between the two proteins. (C) Immunoprecipitation (CoIP) assay to confirm the physical interaction between BACE2 and JAG1. Endogenous BACE2 was immunoprecipitated using a BACE2‐specific antibody to examine its association with JAG1. Normal immunoglobulin G (IgG) was included as the negative control, and input (total cell lysates) served as the positive control. The left panel presents results from T24 BACE2‐overexpressing (OE) cells, and the right panel from parental 5637 cells. (D) Schematic of the experimental workflow: First, cell culture supernatants were collected from T24 EV‐transfected and T24 BACE2‐OE cells. Subsequently, these supernatants were added to 96‐well plates pre‐seeded with naive T24 cells (untreated). Finally, the sensitivity of these supernatant‐treated naive T24 cells to Gemcitabine was determined. (E) CCK‐8 assay assessing gemcitabine sensitivity of recipient T24 cells treated with conditioned medium from T24‐EV or T24‐BACE2‐OE cells in the presence or absence of DAPT. (F) Quantitative real‐time polymerase chain reaction (qPCR) analysis of JAG1 (left panel) and HES1 (right panel) expression in the aforementioned supernatant‐treated cells (*n* = 3 technical replicates). GAPDH was used as the internal reference gene. (G) Western blot analysis to examine JAG1 and HES1 protein expression in the supernatant‐treated cells. GAPDH was used as the loading control. (H) Predicted binding interface and binding sites between JAG1 and BACE2 based on protein–protein docking analysis. JAG1 is shown in blue and BACE2 in yellow. Protein structural information was obtained from the UniProt database. (I) Western blot analysis of BACE2‐mediated JAG1 cleavage in T24 cells. Cell lysates from control, wild‐type BACE2‐expressing, and catalytic‐dead mutant BACE2‐expressing T24 cells were analyzed. The 180 and 25 kDa bands represent full‐length and cleaved JAG1, respectively. GAPDH was used as the loading control. (J) CCK‐8 assay was performed to evaluate gemcitabine sensitivity in control cells, wild‐type BACE2‐expressing cells, and mutant BACE2‐expressing cells. Cell viability was measured after gemcitabine treatment for 48 h. (K) Comparison of BACE1 and BACE2 expression levels in 5637 cells based on RNA‐sequencing data. (L) Colony formation assay evaluating the impact of Verubecestat on cell proliferation. Cells (500/well) were treated with Verubecestat (10, 1, and 0.1 μM) or dimethyl sulfoxide (DMSO) control for 10–14 days. Colonies were fixed, stained, and quantified (*n* = 3). (M) qPCR analysis of JAG1 (left panel) and HES1 (right panel) expression in 5637 cells treated with Verubecestat (10 μM) relative to DMSO‐treated controls (*n* = 3 technical replicates). GAPDH was used as the internal reference gene. (N) CCK‐8 assay assessing the effect of Verubecestat on cell sensitivity to Gemcitabine. Cells (5000/well) were treated with Verubecestat (10, 1, and 0.1 μM) or DMSO control for 48 h with Gemcitabine. Data are presented as mean ± SD. Statistical significance was determined by unpaired two‐tailed *t* tests (F, K, L, M, N). *p ≤ 0.05; **p ≤ 0.01; ***p ≤ 0.001. BACE2, β‐secretase 2; CCK‐8, cell counting kit‐8; DAPT, *N*‐[*N*‐(3,5‐difluorophenacetyl)‐_L_‐alanyl]‐*S*‐phenylglycine *t*‐butyl ester; EV, empty vector; GAPDH, glyceraldehyde‐3‐phosphate dehydrogenase; GRAMM, global range molecular matching; HES1, Hes Family bHLH Transcription Factor 1; IP, immunoprecipitation; JAG1, Jagged1; ns, not significant; SD, standard deviation.

Consistent with this hypothesis, Western blot analysis revealed that BACE2 overexpression in T24 cells led to a marked accumulation of both full‐length JAG1 (~180 kDa) and its ~25 kDa soluble fragment, whereas BACE2 KD yielded the opposite effect (Figure 4A). Protein docking and IP assays supported a physical association between BACE2 and JAG1 (Figure 4B,C). These findings indicate that BACE2 facilitates the generation of JAG1 soluble fragment, suggesting a dual regulatory mechanism involving both direct protein interaction and subsequent pathway activation.

To determine whether Notch signaling in recipient cells is required for the conditioned medium‐mediated paracrine chemoresistance effect, we treated recipient cells with conditioned medium from T24‐EV or T24‐BACE2‐OE cells in the presence or absence of the Notch pathway inhibitor DAPT (Figure 4D). Under T24‐EV‐CM treatment, DAPT did not significantly alter gemcitabine sensitivity. In contrast, T24‐BACE2‐OE‐CM‐induced marked resistance to gemcitabine, and this effect was significantly reversed by DAPT. These results indicate that activation of Notch signaling in recipient cells is required for the paracrine chemoresistance effect induced by conditioned medium from BACE2‐OE cells. Exposure to this conditioned medium significantly reduced gemcitabine sensitivity (Figure 4E) and increased expression of the canonical Notch target HES1 (Figure 4F,G), indicating activation of Notch signaling in a paracrine manner. Notably, conditioned medium treatment also induced endogenous JAG1 expression (Figure 4F,G), suggesting the existence of a positive feedback loop that reinforces sustained Notch pathway activation.

To assess whether BACE2 catalytic activity contributes to JAG1 processing, we compared JAG1 cleavage and gemcitabine response in cells expressing wild‐type BACE2 or the catalytically inactive BACE2‐D303A mutant. Compared with wild‐type BACE2, BACE2‐D303A reduced the generation of the ~25 kDa JAG1 fragment and partially restored gemcitabine sensitivity. These results support that BACE2‐associated JAG1 processing and chemoresistance are dependent, at least in part, on BACE2 catalytic activity (Figure 4H–J).

### 3.5 Verubecestat Enhances Gemcitabine Efficacy In Vitro and In Vivo

On the basis of the above findings showing a cleavage‐dependent BACE2‐JAG1‐Notch signaling axis driving chemoresistance, we next assessed whether pharmacologic inhibition of this pathway could therapeutically reverse this phenotype. Given the dose‐limiting toxicities reported for Notch inhibitors [21, 22], we explored whether targeting the upstream protease BACE2 could represent a more selective strategy to enhance chemotherapy response. To do so, we treated bladder cancer cells with verubecestat, a small‐molecule β‐secretase inhibitor [23]. Although verubecestat can inhibit both BACE1 and BACE2, *BACE2* was expressed at substantially higher levels than *BACE1* in our model system (Figure 4K), providing a rationale for focusing on BACE2 in this context. Nevertheless, because verubecestat is not BACE2‐specific, the pharmacological data should be interpreted as inhibition of BACE‐family protease activity. The BACE2‐dependent contribution is supported by the genetic manipulation and catalytic mutant experiments described above. At 10 μM, verubecestat caused a modest reduction in cell proliferation (Figure 4L), yet markedly reduced *JAG1* production and suppressed expression of the canonical Notch target *HES1* (Figure 4M), indicating effective blockade of downstream signaling. Under these conditions, verubecestat sensitized bladder cancer cells to gemcitabine in a dose‐dependent manner (Figure 4N).

**Figure 5:**
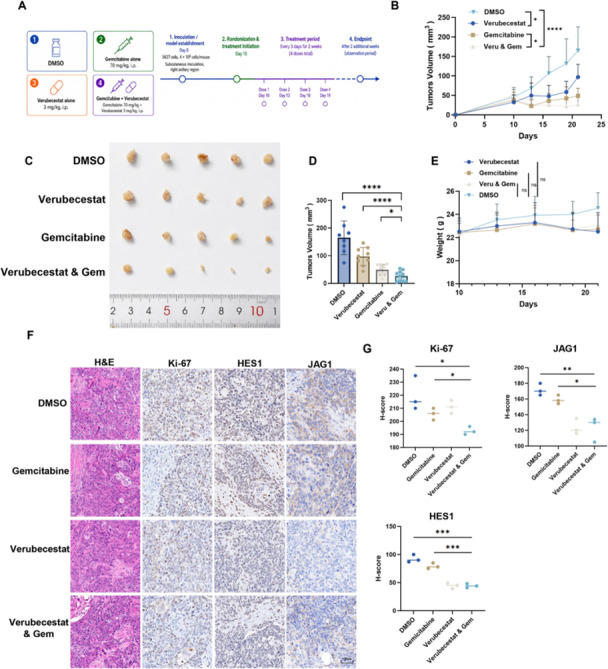
Verubecestat enhances the antitumor efficacy of gemcitabine in a bladder cancer xenograft model. (A) Schematic workflow of the in vivo study. Seven‐week‐old male BALB/c nude mice bearing 5637 cell xenografts were randomized on Day 10 and treated with DMSO, gemcitabine (70 mg/kg), verubecestat (3 mg/kg), or their combination via intraperitoneal injection every 3 days for four cycles. Tumors were harvested on Day 21. (B–D) Tumor growth curves, (B) representative images of excised xenograft tumors at the endpoint (C), and quantification of final tumor volumes (D) in each treatment group. (E) Body weight changes of mice monitored across the indicated time points. (F) Representative images of H&E and immunohistochemical (IHC) staining for Ki‐67, HES1, and JAG1 in paraffin‐embedded tumor sections. Scale bar = 50 μm. (G) Quantification of IHC H‐scores for Ki‐67, HES1, and JAG1 across the treatment groups. Data are presented as mean ± SD. Statistical significance was determined by two‐way repeated‐measures analysis of variance (ANOVA) (B, E) or one‐way ANOVA followed by Tukey's post hoc test (D, G). *p ≤ 0.05; **p ≤ 0.01; ***p ≤ 0.001; ****p ≤ 0.0001. DMSO, dimethyl sulfoxide; H&E, hematoxylin and eosin; HES1, Hes Family bHLH Transcription Factor 1; JAG1, Jagged1; ns, not significant; SD, standard deviation.

We next evaluated therapeutic efficacy in vivo using a mouse xenograft model with four treatment groups: DMSO vehicle control, gemcitabine alone, verubecestat alone, and gemcitabine plus verubecestat. Tumors in the DMSO control group showed continuous growth over the treatment period, whereas verubecestat monotherapy partially delayed tumor growth and gemcitabine alone produced a more pronounced inhibitory effect. Notably, the combination of gemcitabine and verubecestat resulted in the strongest suppression of tumor growth. At the experimental endpoint, the combination group showed a significantly lower tumor volume than the verubecestat monotherapy group and the gemcitabine monotherapy group, indicating that verubecestat further enhanced the antitumor efficacy of gemcitabine in vivo (Figure 5A–D). To preliminarily evaluate treatment tolerability, mouse body weight was monitored throughout the treatment period. Body weight remained generally stable in all treatment groups, including the verubecestat monotherapy group and the gemcitabine plus verubecestat combination group. No significant treatment‐associated body weight loss was observed, suggesting preliminary tolerability of the combination regimen under the tested conditions during treatment (Figure 5E). These results suggest that verubecestat alone or in combination with gemcitabine did not cause significant body weight loss under the tested conditions based on body weight changes under the tested conditions. Consistent with these effects, IHC analysis of excised tumors revealed decreased Ki67 staining, along with reduced JAG1 and HES1 expression, in the combination treatment group (Figure 5F,G), indicating effective suppression of the BACE2‐JAG1‐Notch signaling axis in vivo. Collectively, these findings demonstrate that pharmacologic targeting of BACE2 enhances the antitumor activity of gemcitabine and suggest targeting this proteolytic signaling pathway as a potential strategy to overcome chemoresistance in bladder cancer.

## 4 Discussion

**Figure 6:**
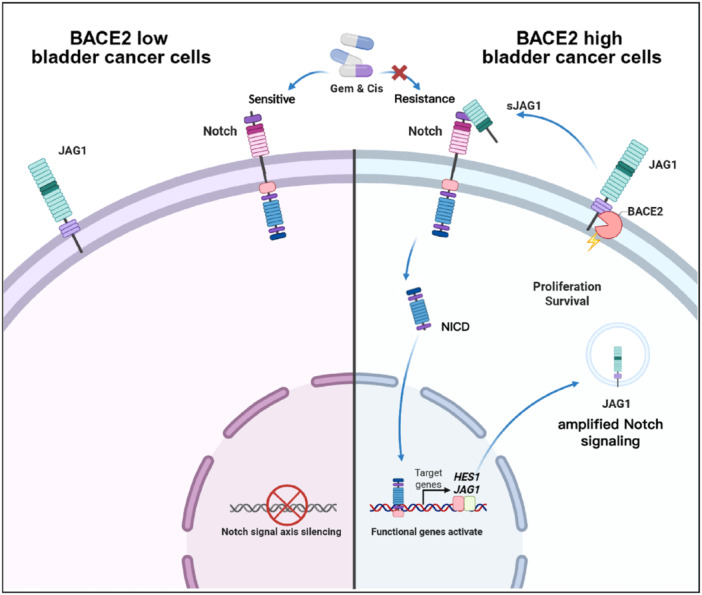
Proposed model of BACE2‐driven chemoresistance in bladder cancer. In BACE2‐low bladder cancer cells (left), the Notch signaling axis remains silenced, rendering cells highly sensitive to chemotherapy. In BACE2‐high cells (right), BACE2 mediates S2 cleavage of transmembrane JAG1 to release soluble JAG1 (sJAG1). Free sJAG1 acts through autocrine and paracrine pathways to bind Notch receptors, triggering receptor cleavage and the release of the Notch intracellular domain (NICD). Upon nuclear translocation, NICD activates downstream target genes, including HES1 and JAG1, forming a positive feedback loop that amplifies Notch signaling, ultimately promoting tumor cell proliferation, survival, and resistance to gemcitabine (Gem) and cisplatin (Cis). Created in BioRender. Lee, G. (2026) https://BioRender.com/pdss9sw. BACE2, β‐secretase 2; HES1, Hes Family bHLH Transcription Factor 1; JAG1, Jagged1.

In this study, we identified *BACE2* as a previously unappreciated regulator of chemoresistance in bladder cancer. By integrating transcriptomic profiles from GemR models with clinical outcome data from TCGA, we prioritized *BACE2* as a candidate associated with poor prognosis. Functional experiments further demonstrated that BACE2 promotes resistance to both gemcitabine and cisplatin, whereas genetic depletion or pharmacologic inhibition of *BACE2* enhances the antitumor effects of chemotherapy. Mechanistically, our data suggest that BACE2 associates with JAG1, promotes the generation of a soluble JAG1 fragment, and enhances JAG1‐Notch signaling, thereby contributing to tumor cell survival under chemotherapeutic stress (Figure 6). Taken together, these findings suggest that BACE2 is not merely associated with adverse clinical outcome but also functionally contributes to treatment resistance and may represent a therapeutically actionable target in bladder cancer.

While previous biochemical studies have established that BACE‐family proteases can cleave JAG1 and activate Notch signaling, the novelty of our work lies in translating this mechanistic axis to the specific context of bladder cancer chemoresistance. First, we identify *BACE2* as a clinically relevant, chemoresistance‐associated molecule in urothelial carcinoma. Furthermore, we demonstrate its functional necessity in driving resistance to standard‐of‐care regimens, including gemcitabine and cisplatin. Mechanistically, our data firmly link the BACE2‐JAG1‐Notch axis to this therapeutically important phenotype. Finally, we provide preclinical proof‐of‐concept that pharmacologic inhibition of this pathway (e.g., via verubecestat) can enhance chemotherapy efficacy. Thus, rather than delineating a fundamentally new enzymatic cleavage event, our findings uncover a previously unrecognized, therapeutically actionable vulnerability in treatment‐refractory bladder cancer.

Our findings also extend the current understanding of Notch signaling in bladder cancer progression and therapy response. The role of Notch signaling in urothelial carcinoma appears to be context dependent. Earlier studies suggested that loss of Notch activity can promote mesenchymal and invasive phenotypes, supporting a tumor‐suppressive role in certain settings [24]. In contrast, subsequent work has identified oncogenic functions for specific Notch pathway components, particularly NOTCH3, which has been linked to poor prognosis and cisplatin resistance in bladder cancer [25]. More recent studies have further associated Notch activation with stem‐like features and chemotherapy‐refractory cell states [26, 27]. Our results are more consistent with this latter model and provide an upstream mechanism for pathway activation. Rather than acting through changes in receptor abundance alone, BACE2 appears to enhance JAG1‐dependent Notch signaling through proteolytic processing of the ligand.

From a therapeutic perspective, targeting BACE2 may offer potential advantages over direct inhibition of the Notch pathway. As an enzyme, BACE2 is more readily amenable to pharmacologic intervention. In addition, targeting an upstream regulator of JAG1 processing may provide a way to suppress aberrant Notch activation without the broader systemic effects associated with direct Notch blockade. Another notable feature of BACE2 inhibition in our study is that it showed limited single‐agent cytotoxicity within the tested range, but substantially improved the response to chemotherapy, supporting its potential utility in combination regimens rather than as monotherapy. At the same time, several issues will need to be addressed before clinical translation. In the revised in vivo experiment, body weight monitoring showed no significant treatment‐associated weight loss in mice receiving verubecestat alone or verubecestat combined with gemcitabine, suggesting preliminary tolerability under the tested conditions. However, body weight monitoring alone does not constitute a comprehensive toxicity assessment. H&E staining of major organs and serum biochemical analyses of hepatic and renal function were not performed in the current study. Therefore, future preclinical studies will be required to more rigorously evaluate the systemic toxicity, therapeutic window, and optimal dosing schedule of verubecestat in combination with chemotherapy. Verubecestat and related β‐secretase inhibitors were not developed specifically for oncology, and their selectivity, tolerability, and optimal scheduling in combination with chemotherapy remain to be defined. It will also be important to determine whether BACE2 expression can serve as a biomarker to identify patients most likely to benefit from this strategy.

Despite these promising findings, our study has several limitations. First, the clinical significance of BACE2 and its association with chemotherapy response were primarily evaluated using retrospective data from publicly available databases (e.g., TCGA and GEO). Prospective, multicenter clinical validation is required to definitively establish its predictive value in patients receiving platinum‐based chemotherapy. Second, our in vivo experiments relied on a subcutaneous xenograft model in immunodeficient mice, which precludes the assessment of BACE2 inhibition within an intact tumor immune microenvironment. Given the complex interplay between chemotherapeutic stress, Notch signaling, and antitumor immunity, future investigations using syngeneic or genetically engineered mouse models are warranted. Although verubecestat effectively reversed chemoresistance with minimal baseline toxicity, its long‐term safety profile, optimal scheduling, and potential off‐target effects in the context of cancer therapy require comprehensive preclinical and clinical evaluation. Finally, while our expression profiling and mutational analyses highlight BACE2 as the predominant functional mediator in our specific models, a minor or context‐dependent contribution from BACE1 to JAG1 processing and chemoresistance cannot be entirely ruled out.

## 5 Conclusion

In conclusion, our study identifies BACE2 as a pivotal driver of chemoresistance in bladder cancer. Through facilitating the proteolytic processing of JAG1 and subsequently hyperactivating Notch signaling, BACE2 orchestrates a cellular state primed to withstand gemcitabine‐ and cisplatin‐induced stress. These findings elucidate a previously unrecognized vulnerability in chemotherapy resistance of bladder cancer, providing a strong mechanistic rationale for repurposing BACE2 inhibitors to restore the clinical efficacy of standard chemotherapeutic regimens.

## Author Contributions

**Zekun Li:** investigation (lead), data curation (lead), methodology (lead), validation (lead), formal analysis (lead), visualization (lead), writing – original draft preparation (lead). **Sanxiang Li:** investigation (equal), methodology (equal), data curation (supporting), writing – review and editing (supporting), validation (equal). **Zhenyu Liu:** visualization (supporting), formal analysis (supporting), software (lead). **Yulin Sun:** writing – review and editing (equal), supervision (equal), funding acquisition (equal). **Yun Zhang:** conceptualization (lead), funding acquisition (lead), writing – review and editing (lead), project administration (lead), resources (lead), supervision (lead).

## Ethics Statement

All animal procedures were reviewed and approved by the Institutional Animal Care and Use Committee of the Cancer Hospital, Chinese Academy of Medical Sciences, under approval number NCC2025A327.

## Consent

The authors have nothing to report.

## Conflicts of Interest

The authors declare no conflicts of interest.

## Data Availability

The data that support the findings of this study are available from the corresponding author upon reasonable request.

## References

[1] F. Bray , M. Laversanne , H. Sung , et al., “Global Cancer Statistics 2022: GLOBOCAN Estimates of Incidence and Mortality Worldwide for 36 Cancers in 185 Countries,” CA: A Cancer Journal for Clinicians 74, no. 3 (2024): 229–263, 10.3322/caac.21834. 38572751

[2] A. G. van der Heijden , H. M. Bruins , A. Carrion , et al., “European Association of Urology Guidelines on Muscle‐Invasive and Metastatic Bladder Cancer: Summary of the 2025 Guidelines,” European Urology 87, no. 5 (2025): 582–600, 10.1016/j.eururo.2025.02.019. 40118736

[3] J. J. de Jong , Y. Lotan , and J. L. Boormans , “Re: EAU Guidelines on Muscle‐Invasive and Metastatic Bladder Cancer,” European Urology 86, no. 5 (2024): 480–481, 10.1016/j.eururo.2024.07.023. 39129108

[4] H. von der Maase , S. W. Hansen , J. T. Roberts , et al., “Gemcitabine and Cisplatin Versus Methotrexate, Vinblastine, Doxorubicin, and Cisplatin in Advanced or Metastatic Bladder Cancer: Results of a Large, Randomized, Multinational, Multicenter, Phase III Study,” Journal of Clinical Oncology 41, no. 23 (2023): 3881–3890, 10.1200/JCO.22.02763. 37549482

[5] Q. Zhang , J. Zhuang , Y. Deng , et al., “miR34a/GOLPH_3_ Axis Abrogates Urothelial Bladder Cancer Chemoresistance *via* Reduced Cancer Stemness,” Theranostics 7, no. 19 (2017): 4777–4790, 10.7150/thno.21713. 29187903 PMC5706099

[6] B. J. Guercio , M. Sarfaty , M. Y. Teo , et al., “Clinical and Genomic Landscape of FGFR3‐Altered Urothelial Carcinoma and Treatment Outcomes With Erdafitinib: A Real‐World Experience,” Clinical Cancer Research 29, no. 22 (2023): 4586–4595, 10.1158/1078-0432.ccr-23-1283. 37682528 PMC11233068

[7] A. Kamoun , A. de Reyniès , Y. Allory , et al., “A Consensus Molecular Classification of Muscle‐Invasive Bladder Cancer,” European Urology 77, no. 4 (2020): 420–433, 10.1016/j.eururo.2019.09.006. 31563503 PMC7690647

[8] J. M. Mota , E. Barnett , J. T. Nauseef , et al., “Platinum‐Based Chemotherapy in Metastatic Prostate Cancer With DNA Repair Gene Alterations,” JCO Precision Oncology 4 (2020): 355–366, 10.1200/po.19.00346. 32856010 PMC7446522

[9] J. Afonso , C. Barbosa‐Matos , R. Silvestre , et al., “Cisplatin‐Resistant Urothelial Bladder Cancer Cells Undergo Metabolic Reprogramming Beyond the Warburg Effect,” Cancers 16, no. 7 (2024): 1418, 10.3390/cancers16071418. 38611096 PMC11010907

[10] C. C. Guo , S. Lee , J. G. Lee , et al., “Molecular Profile of Bladder Cancer Progression to Clinically Aggressive Subtypes,” Nature Reviews Urology 21, no. 7 (2024): 391–405, 10.1038/s41585-023-00847-7. 38321289

[11] R. Vassar , P.‐H. Kuhn , C. Haass , et al., “Function, Therapeutic Potential and Cell Biology of BACE Proteases: Current Status and Future Prospects,” Journal of Neurochemistry 130, no. 1 (2014): 4–28, 10.1111/jnc.12715. 24646365 PMC4086641

[12] Z. Li , K. Fan , C. Suo , et al., “ENO1‐BACE2‐Mediated LDLR Cleavage Promotes Liver Cancer Progression by Remodelling Cholesterol Metabolism,” Journal of Molecular Cell Biology 17, no. 1 (2025): mjaf001, 10.1093/jmcb/mjaf001. 39914449 PMC12246778

[13] F. Farris , V. Matafora , and A. Bachi , “The Emerging Role of β‐Secretases in Cancer,” Journal of Experimental & Clinical Cancer Research: CR 40, no. 1 (2021): 147, 10.1186/s13046-021-01953-3. 33926496 PMC8082908

[14] J. Sáez‐Valero and R. Pérez‐González , “BACE2 Beyond β‐Processing of APP, Its Neuroprotective Role in Cerebrovascular Endothelium,” Journal of Neurochemistry 166, no. 6 (2023): 887–890, 10.1111/jnc.15940. 37587672

[15] K. Chen , Z. Guan , H. Jin , et al., “BACE2‐Induced Aberrant Lymphatic Network Aggravates the Local Inflammation in Arteriovenous Fistulas With Hyperphosphatemia,” Advanced Science 12, no. 43 (2025): e09632, 10.1002/advs.202509632. 40884245 PMC12631814

[16] V. Matafora , A. Elhagh , A. Morelli , et al., “BACE2 Tunes Lipid Uptake Through Lipid Transporters Shedding Supporting Cancer Cell Proliferation,” Journal of Experimental & Clinical Cancer Research: CR 45, no. 1 (2026): 36, 10.1186/s13046-025-03626-x. 41507981 PMC12870435

[17] D. Esterházy , I. Stützer , H. Wang , et al., “Bace2 Is a β Cell‐Enriched Protease That Regulates Pancreatic β Cell Function and Mass,” Cell Metabolism 14, no. 3 (2011): 365–377, 10.1016/j.cmet.2011.06.018. 21907142

[18] G. B. Schulz , S. Elezkurtaj , T. Börding , et al., “Therapeutic and Prognostic Implications of NOTCH and MAPK Signaling in Bladder Cancer,” Cancer Science 112, no. 5 (2021): 1987–1996, 10.1111/cas.14878. 33686706 PMC8088911

[19] W. He , J. Hu , Y. Xia , and R. Yan , “β‐Site Amyloid Precursor Protein Cleaving Enzyme 1 (BACE1) Regulates Notch Signaling by Controlling the Cleavage of Jagged 1 (Jag1) and Jagged 2 (Jag2) Proteins,” Journal of Biological Chemistry 289, no. 30 (2014): 20630–20637, 10.1074/jbc.M114.579862. 24907271 PMC4110275

[20] R. Yan , “Physiological Functions of the β‐Site Amyloid Precursor Protein Cleaving Enzyme 1 and 2,” Frontiers in Molecular Neuroscience 10 (2017): 97, 10.3389/fnmol.2017.00097. 28469554 PMC5395628

[21] C. E. Augelli‐Szafran , H.‐X. Wei , D. Lu , et al., “Discovery of Notch‐Sparing γ‐Secretase Inhibitors,” Current Alzheimer Research 7, no. 3 (2010): 207–209, 10.2174/156720510791050920. 20088802 PMC2858232

[22] A. Mondal , S. Bose , S. Banerjee , and D. Pal , “Role of γ‐Secretase Inhibitors for the Treatment of Diverse Disease Conditions Through Inhibition of Notch Signaling Pathway,” Current Drug Targets 22, no. 15 (2021): 1799–1807, 10.2174/1389450122666210515161312. 33992061

[23] W. Xie , D. Guo , J. Li , et al., “CEND1 Deficiency Induces Mitochondrial Dysfunction and Cognitive Impairment in Alzheimer's Disease,” Cell Death and Differentiation 29, no. 12 (2022): 2417–2428, 10.1038/s41418-022-01027-7. 35732922 PMC9751129

[24] A. Maraver , P. J. Fernandez‐Marcos , T. P. Cash , et al., “NOTCH Pathway Inactivation Promotes Bladder Cancer Progression,” Journal of Clinical Investigation 125, no. 2 (2015): 824–830, 10.1172/jci78185. 25574842 PMC4319408

[25] H. Zhang , L. Liu , C. Liu , et al., “Notch3 Overexpression Enhances Progression and Chemoresistance of Urothelial Carcinoma,” Oncotarget 8, no. 21 (2017): 34362–34373, 10.18632/oncotarget.16156. 28416766 PMC5470974

[26] K.‐J. Wang , C. Wang , L.‐H. Dai , et al., “Targeting an Autocrine Regulatory Loop in Cancer Stem‐Like Cells Impairs the Progression and Chemotherapy Resistance of Bladder Cancer,” Clinical Cancer Research 25, no. 3 (2019): 1070–1086, 10.1158/1078-0432.CCR-18-0586. 30397177

[27] L.‐H. Cheng , C.‐W. Hsu , P.‐M. Yang , et al., “Chemotherapy Resistance of Urinary Bladder Cancer Mediated by a Notch and Wnt Co‐Regulatory Module of Stemness,” Stem Cells 44, no. 4 (2026): sxag002, 10.1093/stmcls/sxag002. 41520149

